# MCH-YOLOv12: Research on Surface Defect Detection Algorithm for Aluminum Profiles Based on Improved YOLOv12

**DOI:** 10.3390/s25175389

**Published:** 2025-09-01

**Authors:** Yuyu Sun, Heqi Yan, Zongkai Shang, Mingxiao Yang

**Affiliations:** College of Computer Science and Technology, Changchun University, Changchun 130022, China; 231502546@mails.ccu.edu.cn (H.Y.); 13386378939@163.com (Z.S.); 19806085316@163.com (M.Y.)

**Keywords:** YOLOv12, deep learning, aluminum profiles, surface defect detection

## Abstract

Surface defect detection in aluminum profiles is critical for maintaining product quality and ensuring efficient industrial production. However, existing detection algorithms often struggle to address the challenges of imbalanced defect categories, low detection accuracy for small-scale defects, and irregular flaw geometries. These limitations compromise both detection accuracy and algorithmic robustness. Accordingly, we proposed MCH-YOLOv12—an improved YOLOv12-based algorithm for precise defect detection. Firstly, we enhanced the original Ghost convolution by incorporating multi-scale feature extraction and named the improved version MultiScaleGhost, which replaced the standard convolutions in the Backbone of YOLOv12. This improvement mitigated the limitations of single-scale convolution, enhancing feature representation and the detection of irregularly shaped defects. Secondly, we addressed the directional and edge-specific nature of defects by enhancing the traditional Channel-wise Gated Linear Unit (CGLU). We proposed the Spatial-Channel Collaborative Gated Linear Unit (SCCGLU), which was embedded after the C3k2 module in the Neck of YOLOv12 to better capture fine-grained features. Finally, we designed a Hybrid Head combining anchor-based and anchor-free detection to improve adaptability to defects of various sizes and shapes. Experimental results on an aluminum profile defect dataset demonstrated improved accuracy, reduced category imbalance, and lower parameters and Floating Point Operations (FLOPs), making the algorithm suitable for real-time industrial inspection.

## 1. Introduction

Aluminum profiles, as an important basic material in the industrial field, have surface defects that directly affect product quality and safety. However, the surface defects of aluminum profiles are characterized by a multi-scale distribution [1], irregular morphology, and strong background interference under complex lighting, which makes it difficult for traditional manual feature-based detection methods to effectively capture the defect features. Furthermore, existing deep learning models still lack proficiency in multi-scale feature fusion, complex background suppression, and adaptability of the anchor frame mechanism to irregular defects [2]. How to improve the algorithm’s multi-scale perception ability and detection robustness for aluminum profile surface defects is a key challenge in the field of industrial vision inspection.

Target detection is one of the core tasks in the field of computer vision [3], aiming to recognize the class of a target in an image or video and locate its position. The technology is widely used in security monitoring, automatic driving, industrial quality inspection, medical imaging, and other fields. With the development of deep learning, target detection algorithms [4] have undergone a revolutionary change from traditional methods to those that are deep neural network-based, the core of which can be divided into two categories: traditional target detection and deep learning-based target detection. The traditional target detection algorithm is the core method in the early computer vision field [5], which mainly describes the texture, shape, and other information of the target by manually designing the features; combines with the sliding window to generate the candidate region; and then utilizes the classifiers, such as Support Vector Machine, Adaboost, etc., to complete discrimination between the target and the background. The process is divided into three stages: candidate region generation, feature extraction, and classification decision. Representative algorithms include the Viola–Jones and Deformable Part-based Model. These algorithms rely on domain knowledge to design features, are computationally lightweight but weak in generalization, and are difficult to cope with multi-scale targets and background interference in complex scenarios. They have gradually been replaced by end-to-end detection algorithms based on deep learning. The deep learning-based target detection algorithm [6] automatically learns image features through a neural network without manually designing features, which significantly improves detection accuracy and efficiency. It is mainly divided into two categories: two-stage algorithms first generate candidate frames through the area suggestion network [7] and then classify and regress them, which is highly accurate but slow; single-stage algorithms predict the target location and category directly on the feature map, which is fast and balanced in terms of accuracy.

In recent years, more and more research work has emerged in the field of surface defect detection of objects. Wang et al. [8] introduced a lightweight de-weighted Bidirectional Feature Pyramid Network (BiFPN) feature fusion structure and an Efficient Channel Attention (ECA) mechanism based on improvements to YOLOv7, and they replaced the bounding box loss function with Scylla-IoU (SIoU) Loss. This approach significantly improved detection accuracy and speed, and the method exhibited superior performance on both the GC10-DET and NEU-DET datasets. However, its robustness under conditions involving very small defects and complex backgrounds still needs improvement. Huang et al. [9] improved the generalization ability of the model for complex defects and rare classes by introducing GAN-based data augmentation and a dynamic anchor frame assignment mechanism into the YOLOv11 framework. This approach achieved high detection accuracy and recall in the PCB defect detection task; however, some misclassification phenomena remained in samples with blurred defect boundaries and small inter-class differences. Chen et al. [10] embedded a Gabor filter layer in Faster R-CNN to suppress fabric background texture interference and used a genetic algorithm to optimize the filtering parameters. This strategy significantly enhanced the model’s ability to locate defects in high-texture interference scenes and achieved a high Mean Average Precision (mAP) in the fabric defect detection task. However, the computational cost was high, which made it difficult to meet the demands of real-time detection. Chen et al. [11] proposed the YOLOv3-dense model based on improvements to YOLOv3. They replaced the backbone network with DenseNet-121 to enhance the detection capability for targets with small defects and complex backgrounds and combined pseudo-defect generation with the Taguchi method for hyperparameter optimization. This approach effectively improved the model’s detection accuracy and generalization capability. The method achieved excellent detection performance across various SMD LED defect categories. However, the complex structure of DenseNet and the high overall computational overhead limited the model’s deployment efficiency in real-time detection tasks. Wang et al. [12] proposed the BL-YOLOv8 model based on improvements to YOLOv8. They enhanced the feature fusion capability and target perception ability by introducing BiFPN and Large Selective Kernel (LSK)-attention. Meanwhile, they replaced the original Spatial Pyramid Pooling-Fast (SPPF) module with Simple Spatial Pyramid Pooling-Fast (SimSPPF) to reduce computational complexity. Experiments on the RDD2022 road defect dataset verified the improvements in the model in terms of accuracy and efficiency. However, issues of missed detections and false detections in cases involving partially blurred cracks and complex backgrounds remained, and the real-time performance of the model slightly decreased. Xu et al. [13] proposed an improved model for tunnel surface defect detection based on enhancements to Mask R-CNN. They enhanced the information propagation capability of low-level features by introducing the Path Aggregation Feature Pyramid Network (PAFPN) and added an edge detection branch to improve the recognition accuracy of defect edges, which significantly boosted the accuracy of both detection and segmentation. This method achieved excellent performance on images of metro tunnel leakage and spalling defects (where leakage refers to water seepage through tunnel walls, and spalling denotes the flaking or breaking off of concrete surfaces), with a 9.18% increase in mAP and an error rate reduced to 0.51%. However, due to the introduction of multiple sub-modules in the model, the overall computational load increased, making it difficult to meet the real-time requirements of low-computing-power devices.

To address the above problems, we proposed an algorithm, MCH-YOLOv12, based on YOLOv12 for detecting surface defects in aluminum profiles. It should be noted that “MCH” stands for “MultiScaleGhost convolution, Channel-wise Gated Linear Unit and Hybrid Head”, which reflects the three core improvements introduced in the proposed model. While preserving the model’s lightweight characteristics, this approach effectively mitigates the issue of defect category imbalance and enhances both the accuracy and robustness in detecting small-scale and irregularly shaped defects. The primary contributions of this research are outlined as follows:

(1) We designed MultiScaleGhost convolution, which enhances the ability to extract multi-scale defect features. This not only improves the detection performance for small targets but also reduces the number of parameters and computational load.
(2) We proposed the SCCGLU. By combining direction-aware channel modeling with edge-guided spatial enhancement, it effectively improved the perception capability for irregular defects. Additionally, we integrated it after the C3K2 module of YOLOv12 using a post-enhancement fusion strategy.
(3) We constructed the Hybrid Head detection head, which integrates the advantages of both anchor-based and anchor-free approaches. This integration thereby enhances the detection accuracy and robustness for irregular defects and defects with class imbalance.

## 2. YOLOv12 Detection Algorithm

YOLOv12 [14], proposed by Yunjie Tian et al., is a real-time object detection algorithm with the attention mechanism at its core. The network architecture of YOLOv12 inherits the classic framework of the YOLO series, comprising three key components: Backbone, Neck, and Head. Specifically, in YOLOv12, the Backbone is tasked with extracting features from the input image [15], thereby providing foundational information for subsequent object detection processes. The Neck serves to fuse and refine the features extracted by the Backbone to enhance feature representability. Finally, the Head performs the ultimate object detection task based on the processed features and outputs both the category and location information of the detected objects.

The Backbone comprises Conv, C3k2, and A2C2f modules. Among these, C3k2 is a module introduced in YOLO11 to assist in feature extraction. The A2C2f, a structure first proposed in YOLO12, is designed for further processing and refinement of features. Its primary function is to extract features from the input image [16], which are then utilized by subsequent network layers for prediction. Notably, its architecture exerts a significant influence on the quality of feature maps generated in the object detection model [17].

The Neck incorporates Concat, Upsample, A2C2f, and C3k2 modules. It fuses and refines the features extracted by the Backbone, integrating multi-level feature information through upsampling and concatenation operations. This process enhances feature expressiveness, renders multi-scale feature fusion more targeted, and provides richer feature information for subsequent detection tasks.

**Figure 1:**
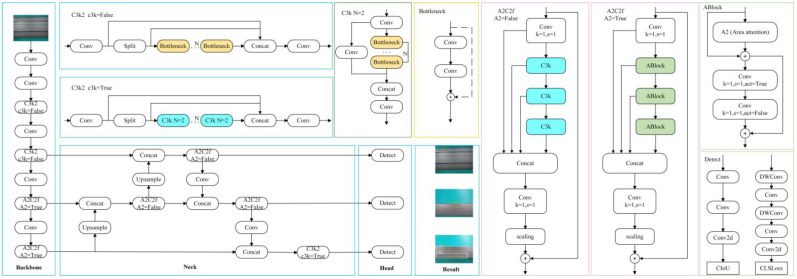
YOLOv12 network structure diagram.

The Head of YOLOv12 shares an identical structure with that of YOLO11 [18], comprising multiple Detect modules. It undertakes the final object detection task and outputs both the category and location information of detected objects based on the features preprocessed by the Backbone and Neck. The network structure of YOLOv12 is shown in Figure 1.

## 3. Methods

YOLOv12 is a state-of-the-art object detection model [19] featuring excellent feature extraction capabilities and detection performance. The authors employed a multi-scale detection architecture to meet the detection requirements of objects with varying sizes. However, in the task of aluminum profile surface defect detection [20], defects often exhibited characteristics such as small target sizes, irregular shapes, and subtle texture variations. Additionally, the images contained significant industrial noise and complex textured backgrounds, which imposed certain limitations on the detection performance of YOLOv12’s original structure in such scenarios. To address these issues, we adopted YOLOv12 as the base model and optimized its structure from three aspects—convolutional modules, attention mechanisms, and detection heads—tailored to the characteristics of aluminum profile surface defect detection [21].

### 3.1. MultiScaleGhost: Lightweight GhostConv Module with Multi-Scale Feature Enhancement

**Figure 2:**
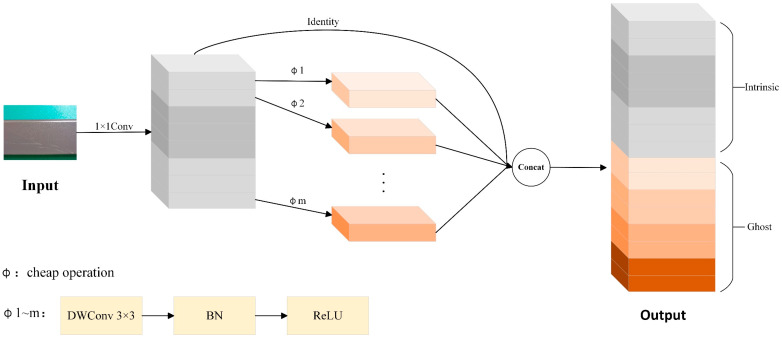
GhostConv structure diagram. In this figure, Φ1 to Φm denote a series of lightweight transformation operations that generate ghost features from the intrinsic feature maps. Each Φ operation is composed of a 3 × 3 Depthwise Separable Convolution (DWConv) [23], followed sequentially by Batch Normalization (BN) [24] and a Rectified Linear Unit (ReLU) [25] activation. By implementing multiple Φ operations in parallel, the module can effectively enrich feature diversity while incurring low computational costs.

Traditional convolution generates feature maps through extensive computations, but studies have shown that many feature maps contain redundancy. GhostConv [22] is a lightweight convolutional architecture that splits feature generation into two parts: one part obtains high-quality features via standard convolution, while the other generates “Ghost features” through inexpensive operations. The structure diagram of GhostConv is shown in Figure 2. However, the original GhostConv employed a fixed linear transformation when generating redundant feature maps, which led to insufficient feature representation in the presence of complex background interference or minor defects, thereby affecting the final detection performance. To address this, we proposed an improved GhostConv module, named MultiScaleGhost.

In Figure 2, the term “cheap operation” denotes lightweight computational operations designed to balance feature extraction effectiveness and computational efficiency. Specifically, within this architecture, it is instantiated as a sequence of DWConv with a 3 × 3 kernel, followed by BN and the ReLU activation function. 

MultiScaleGhost is a multi-scale enhanced version of GhostConv, with its core objective to capture features of targets of different sizes within a lightweight framework. Traditional GhostConv generated derived features via single-scale DWConv, whereas MultiScaleGhost employed multiple convolutional kernels of varying sizes in parallel. This enabled the model to simultaneously focus on local details and global contextual information.

**Figure 3:**
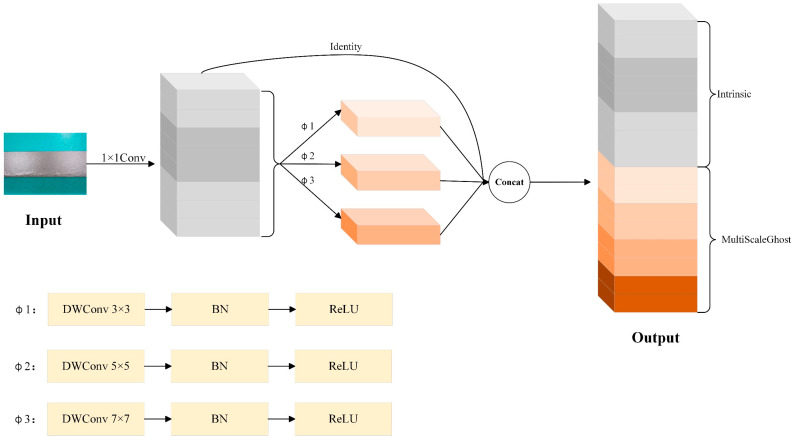
MultiScaleGhost structure diagram. Here, Φ1, Φ2, and Φ3 denote lightweight DWConv operations with distinct kernel sizes (3 × 3, 5 × 5, and 7 × 7, respectively), each followed by BN and ReLU activation. These operations are specifically engineered to generate multi-scale ghost features in an efficient manner.

Specifically, let the input feature map be $X\in R^{C\times H\times W}$. Firstly, the main convolutional module reduced the dimensionality of the input feature map X via a 1 × 1 convolutional layer coupled with BN and the ReLU activation function, generating the preliminary feature representation $X_{p}\in R^{m\times H\times W}$, where $m=\frac{C^{\prime }}{s}$. The specific calculation was Xp=ReLUBNWp⋅X. Then, the multi-scale derivative module employed a parallel branch structure, where each branch utilized DWConv of varying sizes combined with BN and ReLU; the convolution kernel size of each branch was set to $k_{i}=3+2\left(i-1\right)$ (this formula generates a sequence of odd-sized kernels (3 × 3, 5 × 5, 7 × 7, …) to ensure symmetric receptive fields. Starting with $k_{1}=3$ (the smallest odd kernel for fine-grained features), each subsequent kernel increases by 2 (i.e., $k_{i}=k_{i-1}+2$). Substituting $k_{1}=3$, the recursive relation simplifies to $k_{i}=3+2(i-1)$, enabling the achievement of local receptive fields of 3 × 3, 5 × 5, and 7 × 7 across different scales to extract more comprehensive multi-scale feature representations. Each branch generates derived features in the form of Xgi=ReLUBNWgi⋅Xp. Finally, the feature fusion module concatenated the primary feature $X_{p}$ with the derived features $X_{g1},X_{g2},X_{g3},\ldots ,X_{gn}$ generated by each branch along the channel dimension to form the final output feature map $Y\in R^{C^{\prime }\times H\times W}$ (i.e., $Y=Concat\left(X_{p},X_{g1},X_{g2},\ldots ,X_{gn}\right)$). Here, n denotes the number of branches, and the number of channels could be adjusted via an optional 1 × 1 convolution to achieve effective fusion of multi-scale features. The structural diagram of MultiScaleGhost is shown in Figure 3. For ease of reading, we have provided the terminology symbols involved in this section in Table 1.

Compared with the original GhostConv, MultiScaleGhost introduced multi-scale convolution branches while maintaining its lightweight characteristics. Instead of relying solely on simple linear transformations to generate redundant feature maps, it enhanced the ability to perceive defects of different sizes through parallel multi-scale receptive fields. We embedded this module into the Backbone of YOLOv12 to replace the original traditional convolution module. This not only enhances the network’s capability to extract features of surface defects on aluminum profiles but also further improves the model’s detection accuracy and robustness for small-target defects.

### 3.2. Spatial-Channel Collaborative Gated Linear Unit

In object detection networks, an efficient feature representation structure is of great significance for accurately capturing fine-grained information such as small objects and irregularly shaped defects. To enhance the capability of information interaction among feature map channels and suppress redundant features, the Gated Mechanism is widely introduced into deep models. Among these, the Channel-wise Gated Linear Unit is a lightweight and efficient channel-level gating module. It effectively enhances the network’s responsiveness to key channels by segmenting and selectively activating input features along the channel dimension.

#### 3.2.1. Channel-Wise Gated Linear Unit

**Figure 4:**
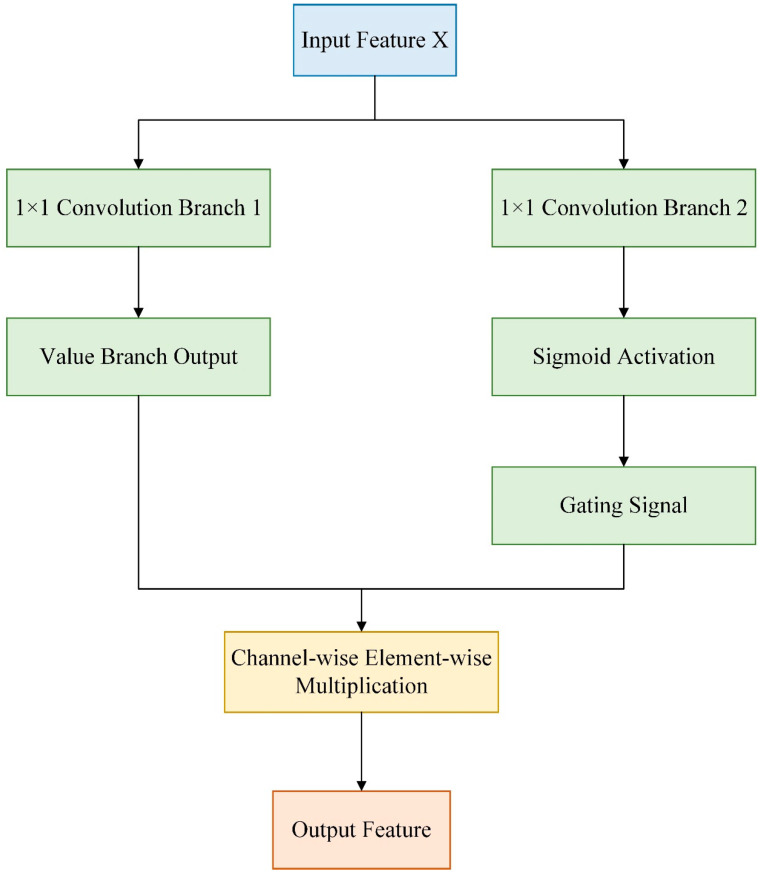
Flowchart of Channel-wise Gated Linear Unit.

The Channel-wise Gated Linear Unit structure was initially extended from the Gated Linear Unit (GLU) [26]. Its core idea is to apply an independent gating mechanism to each channel of the input features, selectively activating or suppressing specific channel information through inter-channel interactions. Unlike the standard GLU, it focused more on feature selection in the channel dimension and was commonly used to enhance the model’s ability to perceive channel semantics. The calculation formula is presented in Equation (1), and the flowchart is illustrated in Figure 4.

(1) $$Channel-wise\;GLU\left(X\right)=Conv_{1}\left(X\right)⊙\sigma \left(Conv_{2}\left(X\right)\right)$$

In the formula, X denotes the input feature map; $Conv_{1}$ and $Conv_{2}$ represent 1 × 1 convolution operations, which generate the value branch and the gating branch, respectively; ⊙ indicates element-wise multiplication, enabling channel-wise selective activation; and σ stands for the Sigmoid activation function.

The original Channel-wise Gated Linear Unit, while capable of assigning different retention weights to each channel via the gating mechanism, thereby enabling the model to focus more on semantically significant channels, had certain structural limitations. The gated features were typically generated solely by simple linear transformations, which made it difficult to model local contextual information. Meanwhile, the independent processing after channel splitting lacked cross-channel information fusion and global dependency modeling. Moreover, this mechanism operated only on the channel dimension, neglecting the importance of spatial structure and local details, which limited its performance in complex visual tasks such as object detection.

#### 3.2.2. Improved Channel-Wise Gated Linear Unit

**Figure 5:**
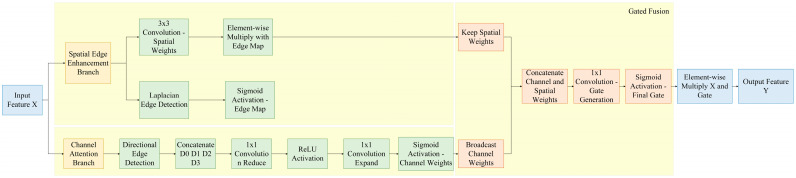
Spatial-Channel Collaborative Gated Linear Unit structure diagram.

To address the aforementioned issues, we proposed the Spatial-Channel Collaborative Gated Linear Unit, which enhanced the targeted capture of defect features through dual-channel collaborative modeling, as illustrated in Figure 5. The core idea of the Spatial-Channel Collaborative Gated Linear Unit was to generate joint gating signals via parallel channel attention branches and spatial-edge enhancement branches, achieving collaborative optimization of channel importance screening and spatial location enhancement. The specific process was as follows: we fed the input feature map $X\in R^{C\times H\times W}$ into two branches simultaneously. The channel attention branch focused on global channel semantic correlations to generate channel-level weights, while the spatial-edge enhancement branch strengthened local spatial features and edge structures to generate spatial-level weights. We fused the outputs of the two branches to obtain a joint gating signal, which was multiplied element-wise with the input features to output the enhanced feature Y.

The channel attention branch adopted the Direction-Aware Channel Attention mechanism to capture the global dependencies among channels while considering the directional characteristics of aluminum profile defects. First, we employed multi-directional edge feature extraction, designing four sets of fixed-direction convolution kernels for the horizontal, vertical, 45°, and 135° directions, respectively. We used these kernels to perform convolution operations on the input features, extracting edge responses in different directions, as shown in Equation (2).

(2) $$D_{i}=W_{dir,i}*X\left(i=0,1,2,3\right)$$

In the formula, $D_{i}$ denotes the edge response feature map in the *i*-th direction, which extracts edge information related to that direction from the input features via a convolution kernel of a specific direction. $W_{dir,i}$ represents the edge detection convolution kernel in the *i*-th direction. These convolution kernels have fixed parameters and do not participate in training; they directly extract edge information in specific directions through predefined gradient operators. ∗ denotes the two-dimensional convolution operation, and X represents the input feature map.

Then, we concatenated the response features from the four directions [D0; D1; D2; D3] and performed dimensionality reduction and mapping via two layers of 1 × 1 convolutions. We generated the channel weights C using the Sigmoid activation function, and the calculation formula is presented in Equation (3).

(3) C=σW2⊗ReLUW1⊗D0;D1;D2;D3

In the formula, C denotes the output channel attention weight; σ represents the Sigmoid activation function; $W_{2}$ signifies the weight parameters of the second 1 × 1 convolutional layer, which maps the intermediate features back to the original number of channels; ⊗ denotes the 1 × 1 convolution operation; $W_{1}$ represents the weight parameters of the first 1 × 1 convolutional layer, which reduces the dimensionality of the concatenated multi-directional edge features; and $\left[D_{0};D_{1};D_{2};D_{3}\right]$ denotes the concatenation of response features from edge detection in four directions along the channel dimension.

To highlight the spatial location information and edge structure of defects, the spatial branch introduced a fusion mechanism of edge detection and spatial attention, and we designed a spatial-edge enhancement branch. First, we extracted the edge response of the input features using the Laplacian Kernel Wedge and obtained the Edge Probability Map E through Sigmoid activation. Its calculation formula is presented in Equation (4).

(4) $$E=\sigma \left(W_{\mathrm{edge}}*X\right),{\;W}_{edge}=\frac{1}{3}\times \left[\begin{array}{ccc} -1 & -1 & -1\\ -1 & 8 & -1\\ -1 & -1 & -1 \end{array}\right]$$

In the equation, E denotes the probability that each spatial position in the input feature X belongs to an edge; σ represents the Sigmoid activation function; $W_{edge}$ signifies the Laplacian operator convolution kernel, which extracts edge information from the input features; ∗ indicates two-dimensional convolution; and X stands for the input features.

Subsequently, we employed a 3 × 3 convolution to perform local spatial modeling on the input features, generating the base spatial weight S. We then fused this weight with the edge probability map to obtain the enhanced spatial attention map S’, and its calculation formula is presented in Equation (5).

(5) $$S^{\prime }=S⊙\left(1+E\right)$$

In the formula, ⊙ denotes element-wise multiplication, and 1+E represents the response weight used to amplify edge regions.

We expanded the channel weight C to the same dimension as the input features, concatenated it with the spatial enhancement weight S’, and then compressed the concatenated result through a 1 × 1 convolution to generate the final gating signal G, thereby achieving adaptive feature selection. Its calculation formula is presented in Equation (6).

(6) $$G=\sigma \left(W_{3}⊗\left[C;S^{\prime }\right]\right)$$

In the formula, σ denotes the Sigmoid activation function; $W_{3}$ represents the weight parameters of the 1 × 1 convolutional layer, which maps the concatenated features back to the original number of channels C; ⊗ denotes the 1 × 1 convolution operation; and $\left[C;S^{\prime }\right]$ signifies the concatenation of the channel attention weight C and the spatial-edge enhancement weight $S^{\prime }$ along the channel dimension.

Ultimately, the module output was the element-wise product of the input features and the gating signal, as presented in Equation (7).

(7) Y=X⊙G

In the formula, Y denotes the output feature after gated enhancement; X represents the input feature of the Spatial-Channel Collaborative Gated Linear Unit; ⊙ indicates element-wise multiplication; and G signifies the joint gating signal.

To justify the employment of the proposed SCCGLU attention module, we performed structural and functional comparisons with several representative attention mechanisms, including the Squeeze-and-Excitation Network (SE-Net) [27], Convolutional Block Attention Module (CBAM) [28], Self-Attention (SA) [29], and Global Attention Module (GAM) [30]. Table 2 summarizes their key differences in terms of attention type, computational complexity, feature enhancement strategies, and application scope.

Compared to existing modules, SE-Net focuses solely on global channel recalibration, while CBAM sequentially applies channel and spatial attention without explicitly modeling edge cues. Although SA and GAM enable stronger global modeling, they introduce substantial computational overhead. In contrast, SCCGLU was specifically designed for industrial surface defect detection, where irregular shapes and blurred edges are prevalent. It incorporates direction-aware channel enhancement and edge-guided spatial refinement, thus achieving a superior balance between effectiveness and efficiency for fine-grained defect localization.

#### 3.2.3. SCCGLU-C3k2 Fusion Structure Design

To balance structural stability and improvement effectiveness, we adopted a post-enhanced fusion strategy, integrating the proposed SCCGLU module after the C3k2 module in the Neck part of YOLOv12. We utilized the C3k2 module as the original feature fusion structure, preserving its capabilities for multi-path convolutional modeling and residual connection-based semantic extraction. Based on the output of the C3k2 module, the SCCGLU module further enhanced the feature map through joint modeling and weighting of both spatial and channel dimensions, thereby improving the model’s responsiveness to fine-grained defects.

**Figure 6:**
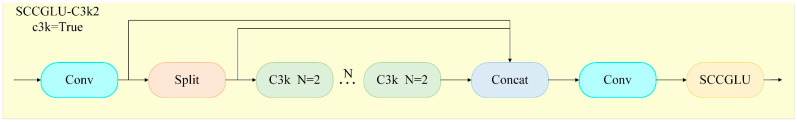
SCCGLU-C3k2 structural diagram.

The specific implementation was as follows: first, we processed the input feature map using the C3k2 module to extract intermediate features. We then fed these intermediate features into the SCCGLU module, which generated channel weights and spatial weights separately before fusing them. Finally, we computed the joint gating signal and used it to perform weighted modulation on the output features of the C3k2 module. The enhanced feature map was denoted as Y=X⊙G, which could be directly connected to the downstream object detection head. By keeping the original YOLOv12 Backbone unchanged, this structure enhanced the feature perception capability of the Neck layer for defect regions and effectively improved detection performance. The structure of SCCGLU-C3k2 is illustrated in Figure 6.

Compared with the original unimproved method, our proposed SCCGLU module achieved fundamental improvements in both structure and function. The original module had certain limitations in capturing the directional and edge features of defects, making it difficult to fully identify defect regions with complex morphologies and blurred boundaries. In contrast, by introducing direction-aware channel modeling, SCCGLU was able to specifically enhance the feature variations of defects in different directions. Meanwhile, combined with an edge-guided spatial enhancement mechanism, it further improved the model’s ability to perceive defects with irregular shapes and blurred edges. Furthermore, we adopted a post-enhancement fusion strategy to integrate it after the C3K2 module of YOLOv12, enabling the enhanced information to fuse more effectively with the backbone features. This significantly improved the detection accuracy and robustness while maintaining computational efficiency.

### 3.3. Hybrid Head: Integrating Anchor-Based and Anchor-Free Approaches

**Figure 7:**
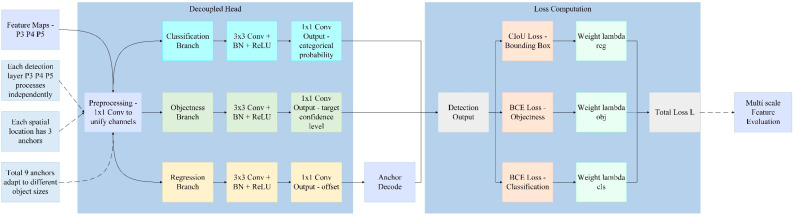
YOLOv12 Detection Head structure diagram.

The detection head of YOLOv12 employs a typical anchor-based [31] design and structurally features a Decoupled Head [32]. Anchor-based object detection represents a prevalent detection paradigm, whose core mechanism involves presetting a set of anchor boxes with varied scales and aspect ratios across the feature map of an image. These anchors are then matched with ground-truth objects to regress both positional offsets and class probabilities. In terms of the loss function, YOLOv12 employed Complete-IoU (CIoU) Loss [33] for bounding box regression and Binary Cross-Entropy (BCE) Loss [34] for object confidence and multi-label classification; the overall loss was a weighted combination of these three components. The structure diagram of the YOLOv12 detection head is illustrated in Figure 7. This detection head structure boasted advantages such as fast inference speed and good convergence. However, it faces challenges in anchor box matching, a process involving the assignment of predefined anchor boxes to ground-truth objects based on overlap criteria, and exhibits inadequate accuracy when detecting small targets, densely packed defects, or surface flaws with complex morphologies.

To further enhance the accuracy and robustness of YOLOv12 in the task of surface defect detection for aluminum profiles, we made targeted improvements to its detection head structure and designed a Hybrid Detection Head based on the fusion of anchor-based and anchor-free approaches. This detection head comprised two parallel branches: the anchor-based branch retained the original structure of YOLOv12, which was used for the detection of conventional targets; the anchor-free branch adopted a dense prediction approach and incorporated a centerness [35] module to enhance the recognition capability for small objects and edge-blurred defects. The two branches, respectively, outputted bounding boxes, confidence scores, and category prediction results. During the training phase, we independently optimized each branch and conducted joint training through a weighted loss combination. During the inference phase, we employed Weighted-NMS [36] to fuse the outputs of the dual branches, thereby enhancing the confidence and localization accuracy of the final prediction boxes. In terms of the loss function, the anchor-based branch adopted CIoU loss and BCE classification loss, while the anchor-free branch combined GIoU regression loss [37], Focal classification loss [38], and BCE centerness loss, forming an overall more discriminative optimization objective function.

#### 3.3.1. Anchor-Free Branch

Anchor-free detection methods eliminate the need for predefined anchor boxes by directly predicting object locations, thereby simplifying model design and reducing computational complexity.

This branch adopted a dense prediction [39] mechanism, directly using each pixel position on the feature map as the center of candidate boxes and regressing the bounding box positions of corresponding targets. In comparison to the anchor box mechanism, which relies on predefined anchor boxes of varying sizes and aspect ratios for object detection, this method eliminates the IoU-based matching process associated with preset anchors, thereby providing enhanced scale adaptability and regression flexibility. It was particularly suitable for locating irregular small target defects.

In the structural design, the anchor-free branch consisted of three parallel sub-modules: BBox Head, Class Head, and Centerness Head. The BBox Head outputted the distance offsets from each pixel to the target boundaries, the Class Head outputted the existence probability for each category, and the Centerness Head learned the geometric distance scores between pixels and the target center, which were used to suppress redundant predictions near the target edges. Ultimately, the total output shape of this branch was $\left[batch,\left(4+1+C\right),H,W\right]$, where 4 corresponds to the four regression parameters of the bounding box (representing the distance offsets from the pixel to the left, right, top, and bottom boundaries of the target, respectively), 1 represented the centerness score, and C represented the number of categories.

**Figure 8:**
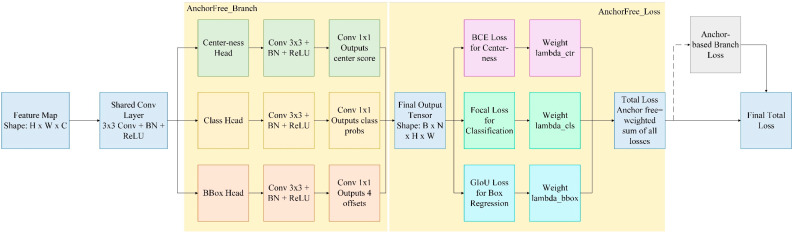
Flowchart of the anchor-free branch.

In terms of the loss function, we employ GIoU Loss for bounding box regression to enhance the constraint on the geometric alignment of predicted boxes. To address the class imbalance problem, where certain object categories have significantly more training instances than others and potentially bias the model toward dominant classes, we adopted Focal Loss for classification. Additionally, BCE Loss is used as the supervision signal for centerness prediction. We jointly optimized this branch with the anchor-based branch, which effectively enhanced the detection capability for tiny, dense, and irregularly shaped defects. The specific process is illustrated in Figure 8.

Although the introduction of an additional anchor-free branch may appear to increase computational load, it actually simplifies several stages of the detection pipeline. Specifically, anchor-free detection eliminates the need for generating and matching a large number of predefined anchors, as well as the computationally expensive IoU-based anchor-matching process during inference. The dense prediction strategy allows each feature map location to directly contribute to the prediction without relying on predefined priors, leading to a more streamlined and efficient detection process. Consequently, the overall inference efficiency remains high, ensuring that the model maintains real-time performance while achieving improved robustness and accuracy.

#### 3.3.2. Balancing and Optimization of Multiple Loss Functions in Hybrid Head

During the training process, multiple loss functions were employed to optimize the performance of the Hybrid Head. To balance the contributions of these loss functions, a rigorous weight assignment and tuning strategy was devised. The total loss $L_{total}$ was defined as a weighted sum of individual loss functions: $L_{total}=\lambda_{1}L_{CIoU}+\lambda_{2}L_{BCE-cls}+\lambda_{3}L_{GIoU}+\lambda_{4}L_{Focal}+\lambda_{5}L_{BCE-center}$ Here, $L_{CIoU}$ and $L_{GIoU}$ serve for bounding box regression, aiming to enhance the accuracy of object localization. $L_{BCE-cls}$ and $L_{Focal}$ focus on classification tasks, ensuring that the model can accurately identify object categories. $L_{BCE-center}$ is utilized to control prediction quality and reduce the occurrence of low-quality predictions. The coefficients $\lambda_{1}$ to $\lambda_{5}$ determine the relative importance of each loss function.

To determine these weight coefficients, a grid search combined with validation set feedback was adopted. Initially, uniform weights $\left(\lambda_{1}=\lambda_{2}=\cdots =\lambda_{5}=1\right)$ were set as a baseline. Subsequently, each $\lambda_{i}$ was systematically varied within a reasonable range (e.g., $\lambda \in \left[0.1,5\right]$, with a step size of 0.5), and key metrics on the validation set, such as mAP for detection accuracy and inference speed for efficiency, were monitored. For example, increasing the weight of $L_{BCE-center}$ (i.e., increasing $\lambda_{5}$) was observed to suppress low-quality predictions but risked over-penalizing edge cases. Thus, it was balanced against $\lambda_{1}$ (weight for $L_{CIoU}$) to maintain the stability of bounding box regression. After multiple rounds of iterative adjustments, the final weight combination was determined as $\lambda_{1}=1.5$, $\lambda_{2}=1.0$, $\lambda_{3}=1.2$, $\lambda_{4}=2.0$, and $\lambda_{5}=0.8$. This combination achieves a favorable balance among precise object localization, robust classification performance, and high-quality prediction filtering.

Moreover, the weight tuning strategy was based on a deep understanding of the objectives of each loss function. As regression losses, $L_{CIoU}$ and $L_{GIoU}$ were assigned moderate weights to ensure the accuracy of bounding box localization, which is crucial for object detection tasks. $L_{\mathrm{Focal}}$, as the classification loss for the anchor-free branch, was given a relatively high weight $\lambda_{4}=2.0$ to address the foreground–background imbalance issue. In contrast, $L_{BCE-center}$ was assigned a relatively low weight ($\lambda_{5}=0.8$) to avoid over-suppressing valid but off-center predictions. This allows it to assist regression losses, thereby improving overall performance without overly constraining the model.

This balancing and tuning strategy for loss functions adheres to the principles of multi-task learning optimization. That is, loss weights are assigned based on the relative difficulty and importance of subtasks (localization, classification, and quality control), thereby enabling the Hybrid Head architecture to operate efficiently in complex object detection tasks.

#### 3.3.3. Weighted Non-Maximum Suppression

During the inference phase, we employed the Weighted Non-Maximum Suppression (Weighted-NMS) method to integrate predictions from the anchor-based and anchor-free approaches, thereby further enhancing the confidence and localization accuracy of the final prediction boxes. First, we preliminarily filtered the sets of candidate bounding boxes output by the two branches separately. Then, we merged the two sets of candidate bounding boxes by category and performed weighted fusion on candidate bounding boxes with higher overlap. The position of the fused prediction box was obtained by weighted averaging the bounding box coordinates of multiple candidate boxes according to their confidence scores, and the confidence score was the weighted sum of the confidence scores of the participating candidate boxes. The calculation formulas are presented in Equations (8) and (9).

(8) $$\overset{\wedge }{B}=\frac{\sum_{i=1}^{n}s_{i}\cdot B_{i}}{\sum_{i=1}^{n}s_{i}}$$

(9) $$\overset{\wedge }{s}=\frac{\sum_{i=1}^{n}s_{i}^{2}}{\sum_{i=1}^{n}s_{i}}$$

In the equations, $B_{i}$ denotes the position of the *i*-th candidate box; $s_{i}$ represents its corresponding confidence score; and $\overset{\wedge }{B}$ and $\overset{\wedge }{s}$ indicate the fused box position and confidence score, respectively.

**Figure 9:**
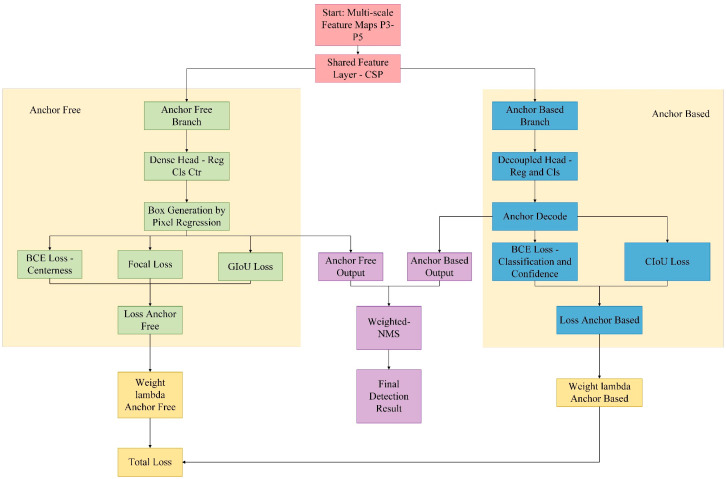
The flowchart of the Hybrid Head detector with anchor-based and anchor-free.

This strategy effectively integrated the high recall rate of the anchor-based branch with the precision advantage of the anchor-free branch in small object detection, removed redundant boxes, and retained high-quality prediction results. The flowchart of the Hybrid Head detector with anchor-based and anchor-free approaches is shown in Figure 9.

Compared with the detection head before improvement, our proposed Hybrid Head achieved a fundamental breakthrough in both structural design and detection mechanism. Traditional anchor-based detection heads relied on priors for target localization. Although they exhibited high localization accuracy, they were prone to limitations imposed by prior box designs when confronted with targets of irregular shapes or class imbalance. In contrast, anchor-free methods performed regression via key points or center points, exhibiting stronger generalization ability and higher adaptability. However, they might suffer from localization deviations in the case of small targets or complex backgrounds.

Our proposed Hybrid Head detection head integrates the two methods into a unified framework. It retained the advantages of anchor-based methods in precise regression while incorporating the robustness of anchor-free methods in adapting to complex morphologies and category distributions. Through the complementary synergy of this structure, the model exhibited higher accuracy and stability in detecting irregular defects and class-imbalanced data, significantly outperforming traditional detection heads that solely utilized a single detection mechanism.

Table 3 summarizes the loss functions used in this subsection, including their target modules, purposes, and distinguishing characteristics, which clarify their roles in the training process.

**Figure 10:**
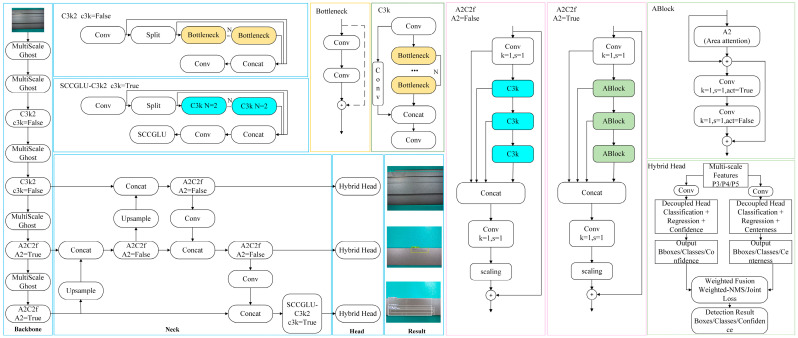
MCH-YOLOv12 network architecture diagram.

We proposed three improvements based on YOLOv12: first, we designed a lightweight convolutional module with multi-scale feature enhancement, named MultiScaleGhost, to replace the original Conv layer in the Backbone, thereby enhancing feature extraction capability and efficiency. Second, we proposed a Spatial-Channel Collaborative Gated Linear Unit, which achieved significant enhancement of fine-grained defect regions through the collaborative fusion of direction-aware channel attention and edge-guided spatial modeling. Using a post-enhancement strategy, we integrated this unit after the C3k2 module in the Neck section of YOLOv12. Third, we constructed a Hybrid Detection Head that integrates anchor-based and anchor-free detection methods, combining the advantages of both approaches to enhance localization accuracy and sample adaptability. The overall improved MCH-YOLOv12 network structure is illustrated in Figure 10.

To provide a clearer overview of the architectural modifications, we present a comparative table (Table 4) summarizing the structural differences between the original YOLOv12 and MCH-YOLOv12. This table highlights the specific components that were modified, added, or replaced, along with the rationale behind each modification.

## 4. Experiments

### 4.1. Dataset

**Figure 11:**
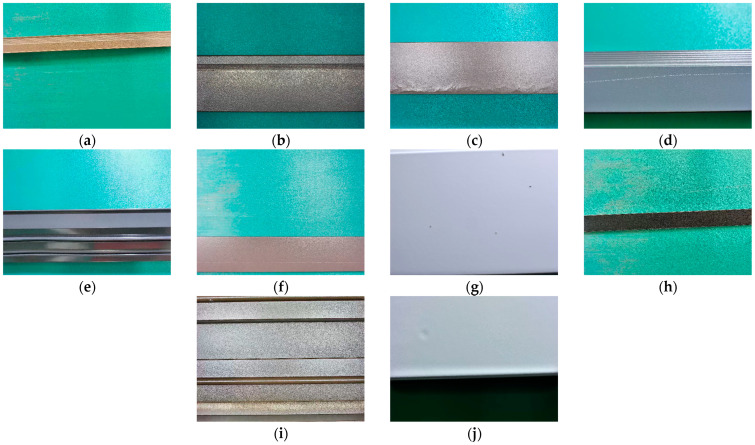
Ten types of defects on the surface of aluminum profiles. (**a**) An image containing the defect known as jupi. (**b**) An image containing the defect known as budaodian. (**c**) An image containing the defect known as tufen. (**d**) An image containing the defect known as cahua. (**e**) An image containing the defect known as aoxian. (**f**) An image containing the defect known as qikeng. (**g**) An image containing the defect known as zangdian. (**h**) An image containing the defect known as tucengkailie. (**i**) An image containing the defect known as loudi. (**j**) An image containing the defect known as pengshang.

The images in this dataset were sourced from the preliminary open dataset of the “Aluminum Profile Surface Defect Recognition” competition, which was part of the 2018 Guangdong Industrial Intelligent Manufacturing Big Data Innovation Contest—Intelligent Algorithm Challenge [40]. The preliminary round of the competition was a classification contest, and its corresponding dataset contained only classification labels. We annotated defects in the original classification dataset, reconstructing it into a defect detection dataset. To construct the detection dataset, we re-annotated the original classification images using the LabelImg tool (version 1.8.4), drawing bounding boxes around visible defect regions. To ensure labeling quality, each annotated image was reviewed by at least two annotators. Discrepancies were resolved through cross-checking and consensus discussions, with the aim of maintaining consistency and accuracy across the dataset. We named this annotated detection dataset APDDD. The images in the APDDD dataset had a resolution of 2560 × 1920 and contained ten types of defects: aoxian (Dent), budaodian (Non-conductive Area), cahua (Scratch), jupi (Orange Peel), loudi (Exposed Substrate), pengshang (Impact Damage), qikeng (Pit), tufen (Powder Bulge), tucengkailie (Coating Crack), and zangdian (Dirt Spot). The dataset consisted of a total of 1885 images, which we divided into training, validation, and test sets in a 7:2:1 ratio. Specifically, the training set contained 1320 images, the validation set included 377 images, and the test set comprised 188 images. Examples of the ten types of aluminum profile surface defects are illustrated in Figure 11.

### 4.2. Experimental Environment and Parameter Settings

This study was implemented on the Ubuntu system using the PyTorch deep learning framework and Python programming environment, with specific experimental configurations detailed in Table 5. During the training process, we set the parameters shown in Table 6.

### 4.3. Evaluation Metrics

We employed evaluation metrics, including precision, recall, mAP@0.5, mAP@0.5~0.95, parameters, and Floating Point Operations (FLOPs). Among these, precision primarily measured the proportion of instances predicted as positive by the model that were actually positive. Recall reflected the comprehensiveness and coverage of the model in object detection; mAP could comprehensively reflect the model’s detection performance across different categories. Parameters served as a crucial metric for assessing model complexity and computational requirements. FLOPs represented the number of floating-point operations required during the model’s forward propagation, which objectively reflected the model’s computational complexity. The specific calculation formulas are presented in Equations (10)–(12).

(10) $$Precision=\frac{TP}{TP+FP}$$

(11) $$Recall=\frac{TP}{TP+FN}$$

(12) $$mAP=\frac{\sum_{n=0}^{N-1}\int_{0}^{1}Precision\left(Recall\right)d\left(Recall\right)}{N}$$

In the formulas, TP represents the number of positive samples correctly identified as positive; FP represents the number of negative samples incorrectly classified as positive; FN represents the number of positive samples incorrectly classified as negative; n denotes the category index; N indicates the total number of detection categories.

### 4.4. Comparison with YOLOv12

To verify the improved detection performance of MCH-YOLOv12, we conducted comparative experiments between MCH-YOLOv12 and YOLOv12n. Table 7 presents the precision of MCH-YOLOv12 and YOLOv12n for each defect type, as well as the mAP@0.5 for all defects. The experimental results indicated that MCH-YOLOv12 achieved a 3.5% improvement in mAP@0.5, with varying degrees of enhancement in precision for each defect type. Specifically, the precision for qikeng, zangdian, and loudi increased by 8.2%, 6.7%, and 9.8%, respectively. These results demonstrate that the improved model can effectively address the class imbalance issue in defect detection and enhance detection accuracy.

**Figure 12:**
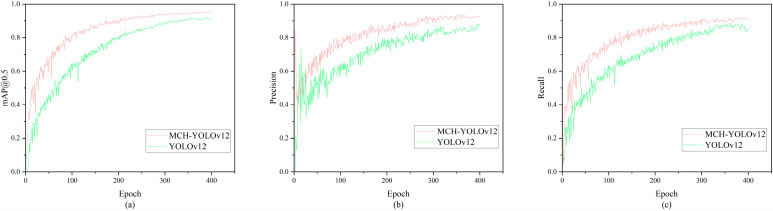
Variation curves of evaluation metrics. (**a**) Comparison of mAP@0.5 between YOLOv12 and MCH-YOLOv12; (**b**) comparison of precision between YOLOv12 and MCH-YOLOv12; (**c**) comparison of recall between YOLOv12 and MCH-YOLOv12.

Figure 12 illustrates the variation curves of several key evaluation metrics for MCH-YOLOv12 and YOLOv12n during the training process. As observed in the figure, after 10 epochs of training, the values of mAP@0.5, precision, and recall were all significantly better than those of the baseline. After 300 epochs of training, the model tended to stabilize. Compared to the baseline, our proposed improvement method demonstrated better training effectiveness and superior detection performance.

**Figure 13:**
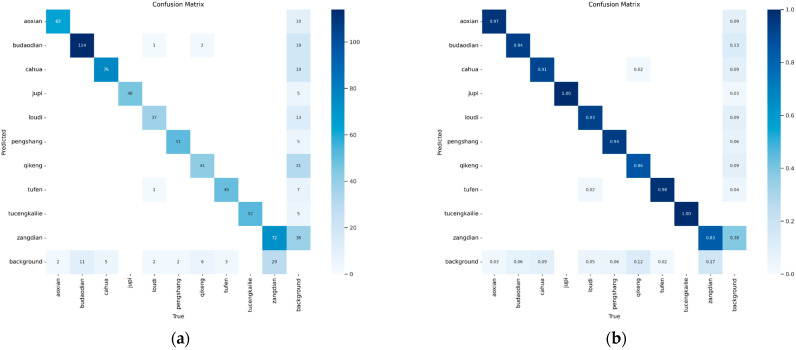
Comparison of Confusion Matrices between YOLOv12 and MCH-YOLOv12. (**a**) The Confusion Matrix of YOLOv12; (**b**) the Confusion Matrix of MCH-YOLOv12.

To more comprehensively evaluate the model’s ability to identify various types of defects, we introduced a confusion matrix for visual analysis, comparing the classification performance of the model before and after improvements, as shown in Figure 13. In the graph, the horizontal axis represents the true class, the vertical axis represents the model’s predicted class, and the darker the color, the higher the prediction accuracy for that class.

As observed in the figure, MCH-YOLOv12 demonstrated higher recognition accuracy across most defect categories. Particularly in categories such as jupi and tucengkailie, the prediction results were almost flawless, with an accuracy rate of 1.00 on the diagonal. This indicated that the model was more robust in extracting and identifying features of these defect types. Furthermore, categories such as qikeng, cahua, and pengshang, which previously had higher misdetection rates, also showed significant improvements in accuracy. This indicated that the enhanced model could more effectively distinguish these easily confused categories.

### 4.5. Ablation Experiment

To validate the independent contributions and combined effects of each improvement module on detection performance, we designed a series of ablation experiments. We progressively introduced each module into the original YOLOv12 and analyzed their effectiveness through performance comparisons. The experiments used the original YOLOv12 as the baseline (A0), sequentially adding MultiScaleGhost (A1), SCCGLU-C3K2 (A2), Hybrid Head (A3), and various module combinations (A4~A7). We maintained consistent training strategies and datasets, introduced only structural modifications, and ensured the fairness of the experiments. The experimental results are presented in Table 8.

**Figure 14:**
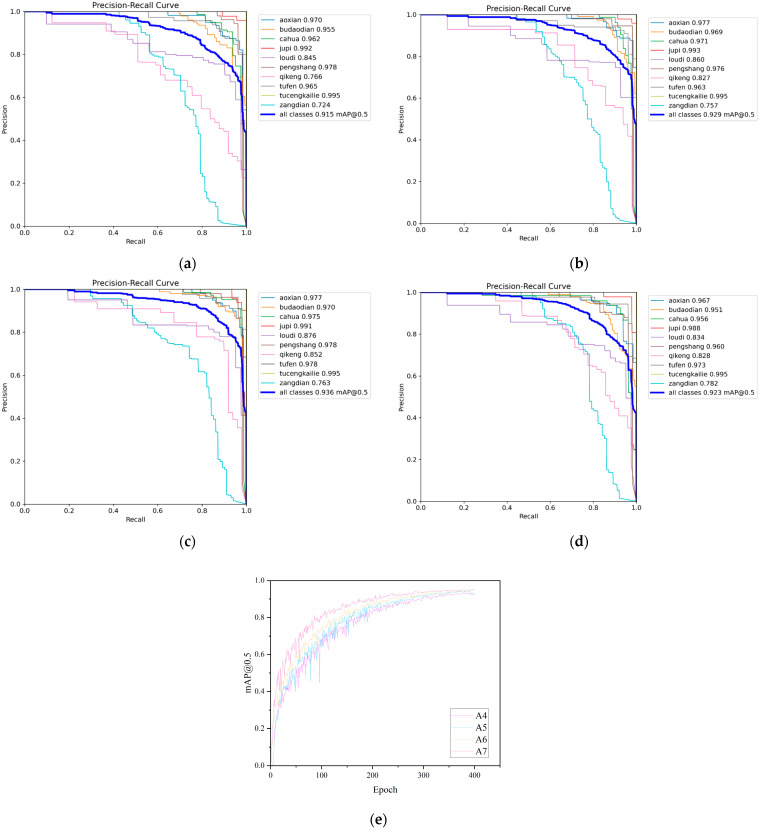
Visualization results of the ablation experiments. (**a**) The precision–recall curve of A0; (**b**) the precision–recall curve of A1; (**c**) the precision–recall curve of A2; (**d**) the precision–recall curve of A3; (**e**) the mAP@0.5 comparison chart of A4~A7.

As observed in the table, all three improvements positively impacted the model’s detection accuracy, validating their respective effectiveness. Among them, A3 delivered the most significant performance improvement, demonstrating its strong expressive capability in locating and identifying multi-scale defect targets. A1 achieved improved accuracy while reducing the number of parameters, which demonstrated that its lightweight design maintained strong feature extraction capabilities without compromising efficiency. The improvement of A2 on mAP@0.5~0.95 was particularly significant, indicating that the proposed Spatial-Channel Collaborative Gating Mechanism helped enhance the model’s perception ability of object boundaries under different IoU thresholds, thereby improving overall robustness. The final integrated A7 model, which incorporated all improvements, achieved well-balanced performance in detection accuracy, parameter control, and computational load. This fully demonstrated the synergistic benefits among its modules. The visualization results of models A0 to A7 are presented in Figure 14.

The experimental results demonstrated that the A1 model had enhanced capability in capturing multi-scale features, and its mAP@0.5 increased to 0.929. Notably, the PR curve for small target defects shifted toward the upper-left corner of the coordinate system, indicating that multi-scale feature fusion had effectively improved the issues of missed detection and false detection of small defects. Leveraging the spatial-channel collaborative gating mechanism, the A2 model enhanced the model’s ability to distinguish similar defects, achieving an mAP@0.5 of 0.936. The dispersion of PR curves across various categories was reduced, validating the optimization effect of collaborative gating on fine-grained feature differentiation. The precision retention capability of the A3 model at different recall rates was further enhanced, with an mAP@0.5 of 0.932. Its robustness in detecting irregular and polymorphic defects improved, and the overall coverage area of the PR curve expanded. The progressive changes in the aforementioned PR curves intuitively demonstrated the innovative value of MultiScaleGhost in enhancing feature extraction, SCCGLU-C3k2 in optimizing feature interaction, and Hybrid Head in balancing the detection needs for different defects. The collaborative efforts of various modules enhanced the accuracy and robustness of the model in the task of surface defect detection for aluminum profiles, providing a superior solution for defect detection in industrial scenarios.

### 4.6. Comparative Experiment

To comprehensively validate the overall performance of the proposed MCH-YOLOv12 model in the task of surface defect detection for aluminum profiles, we designed a large-scale comparative experiment. We selected mainstream versions of the YOLO series (YOLOv5, YOLOv7, YOLOv8, YOLOv9, YOLOv10, and YOLOv11), as well as Faster-RCNN [41] and Single Shot MultiBox Detector (SSD) [42] as reference objects, covering the typical development stages of object detectors in recent years. These models exhibited distinct characteristics in detection accuracy, speed, and model architecture, serving as backbone frameworks widely adopted in practical application scenarios such as industrial inspection and unmanned inspection. By conducting comparisons under a unified experimental setup, we could more intuitively evaluate the applicability and advantages of the proposed method in practical tasks.

**Figure 15:**
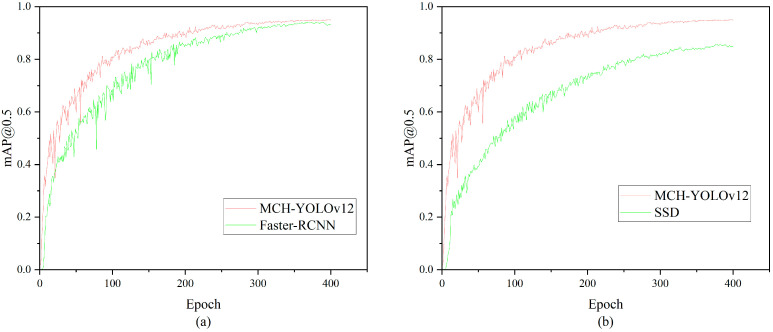
Comparison chart of mAP@0.5. (**a**) The comparison results of mAP@0.5 between MCH-YOLOv12 and Faster-RCNN. (**b**) The comparison results of mAP@0.5 between MCH-YOLOv12 and SSD.

**Figure 16:**
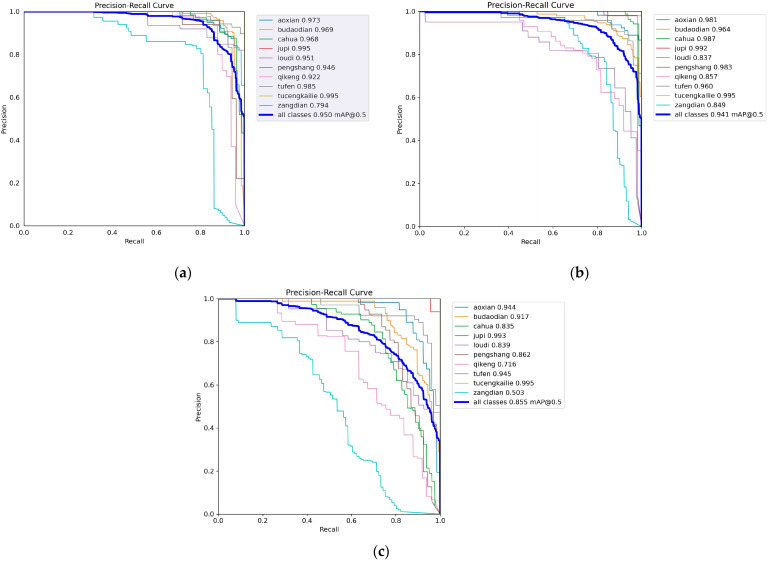
Visualization result diagram. (**a**) The precision–recall curve of MCH-YOLOv12. (**b**) The precision–recall curve of Faster-RCNN. (**c**) The precision–recall curve of SSD.

The experimental results are presented in Table 9. As a representative of lightweight architectures, YOLOv5 had certain advantages in inference speed but lagged relatively in detection accuracy. YOLOv7 and YOLOv8 performed well on the mAP metric; particularly, YOLOv8 achieved superior overall performance after introducing the dynamic Head design. YOLOv9 to YOLOv11 exhibited an increasing trend in accuracy, but this was accompanied by a continuous rise in the number of parameters and computational requirements, which led to higher deployment costs. Faster-RCNN achieved excellent detection performance, particularly in mAP@0.5~0.95, where it ranked the highest among all detection models, surpassing MCH-YOLOv12 by 2.4%. However, its FLOPs and parameters were approximately 15 times and 6 times those of MCH-YOLOv12, respectively. Despite maintaining large FLOPs and high parameter demands, SSD yielded suboptimal detection results. The comparative results between MCH-YOLOv12, Faster-RCNN, and SSD are presented in Figure 15 and Figure 16. In comparison, the MCH-YOLOv12 model proposed in this paper achieved the highest mAP@0.5 and mAP@0.5~0.95 while maintaining a low parameter count and computational cost. This demonstrated its strong detection capabilities and engineering deployability.

Figure 16a–c, respectively, present the precision–recall curves of MCH-YOLOv12, Faster R-CNN, and SSD models on the task of aluminum profile surface defect detection. A comprehensive comparison showed that MCH-YOLOv12 exhibited the most superior detection performance across all defect categories, achieving an overall mAP@0.5 of 95.0%, which was significantly higher than those of Faster-RCNN (94.1%) and SSD (85.5%). This demonstrated its stronger precision and recall capabilities. Based on the trends of the PR curves, the curves of MCH-YOLOv12 for most categories were smooth and close to the upper-left corner, indicating its strong discriminative ability for different types of defects. Faster-RCNN performed second best; although it exhibited outstanding performance in some categories, its overall stability was slightly inferior. The PR curves of SSD generally declined early, reflecting significant shortcomings in handling small targets and complex backgrounds. Thus, MCH-YOLOv12 demonstrated the optimal detection performance while maintaining a high inference speed, making it particularly suitable for industrial vision scenarios where high precision and robustness are required.

**Figure 17:**
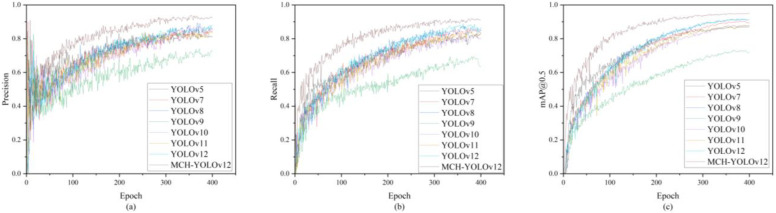
Visualization results of the comparative experiments. (**a**) The precision comparison chart of each model; (**b**) the recall comparison chart of each model; (**c**) the mAP@0.5 comparison chart of each model.

As observed in the table, MCH-YOLOv12 achieved a 7.8% improvement in mAP@0.5 compared to YOLOv5 and a 7.0% improvement compared to the current mainstream version, YOLOv11. On the more stringent mAP@0.5~0.95 metric, the improvement was also significant, with a 6.8% increase over YOLOv11. It is noteworthy that although YOLOv11 featured a larger parameter scale and higher computational complexity, its improvement in detection accuracy tended to plateau. In contrast, the method proposed in this paper achieved a favorable balance between lightweight design and accuracy through module optimization and structural reconstruction. This makes it particularly suitable for defect detection tasks in industrial scenarios where both real-time performance and accuracy are highly needed. The visualization of the comparative experimental results is presented in Figure 17.

**Figure 18:**
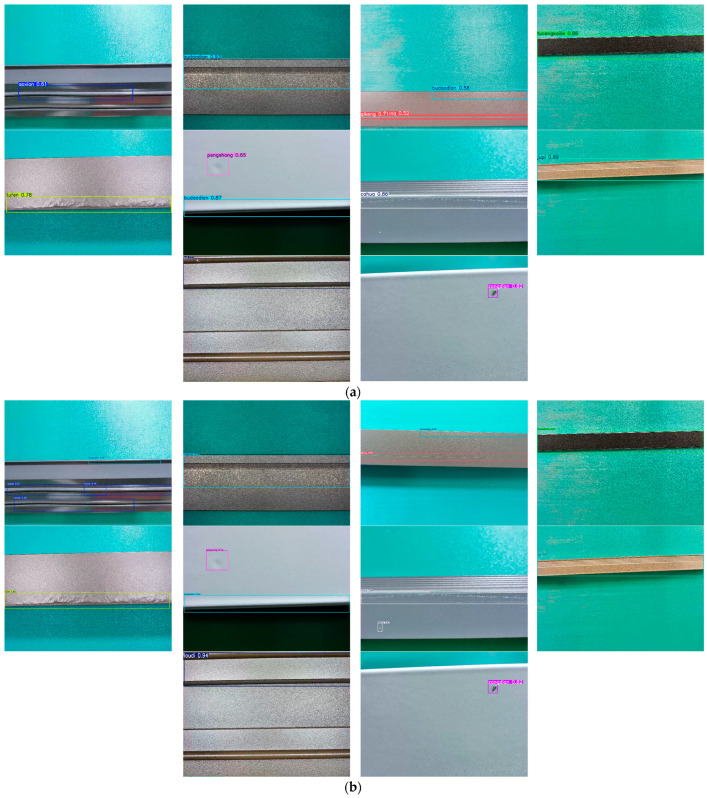
Performance of 10 types of surface defects on aluminum profiles in YOLOv12 and MCH-YOLOv12. (**a**) Detection results of the 10 defects in YOLOv12. (**b**) Detection results of the 10 defects in MCH-YOLOv12.

The detection results of ten types of surface defects on aluminum profiles using YOLOv12 and MCH-YOLOv12 are presented in Figure 18.

As observed in the figure, MCH-YOLOv12 exhibits superior detection performance compared to the original YOLOv12 across the ten types of defects on aluminum profiles.

### 4.7. Generalization Evaluation on the NEU-DET Dataset

Although the proposed MCH-YOLOv12 model demonstrated excellent performance on the aluminum profile defect dataset, its generalizability to other types of surface defects remained to be validated. Therefore, to further demonstrate the robustness and applicability of our method, we conducted additional experiments on the publicly available NEU-DET dataset.

**Figure 19:**
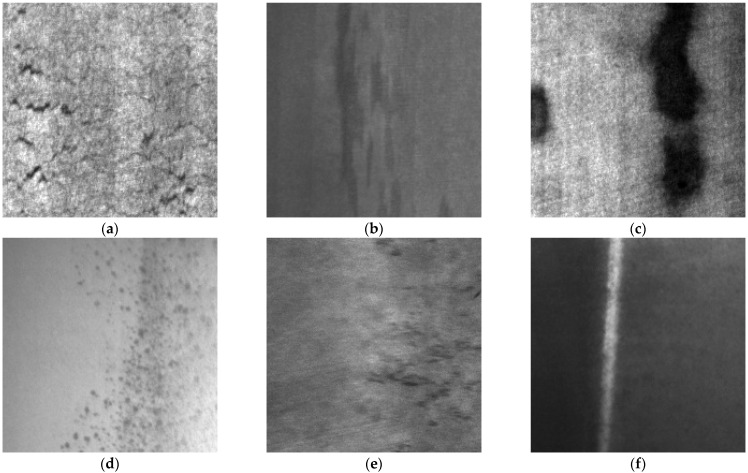
The six types of defects in the NEU-DET dataset. (**a**) An image containing the defect known as crazing. (**b**) An image containing the defect known as inclusion. (**c**) An image containing the defect known as patches. (**d**) An image containing the defect known as pitted surface. (**e**) An image containing the defect known as rolled-in scale. (**f**) An image containing the defect known as scratches.

To verify the generalization capability of our method, we further evaluated the model on the widely used public NEU-DET dataset. This dataset, constructed by the research team led by Kechen Song at Northeastern University, contained 1800 grayscale images of 200 × 200 pixels, evenly distributed across six common steel surface defect categories: crazing, inclusion, patches, pitted surface, rolled-in scale, and scratches, with 300 labeled samples per class. This balanced dataset provided a solid benchmark for comprehensively evaluating defect detection models under diverse defect types and imaging conditions. The six types of defects in the NEU-DET dataset are shown in Figure 19.

**Figure 20:**
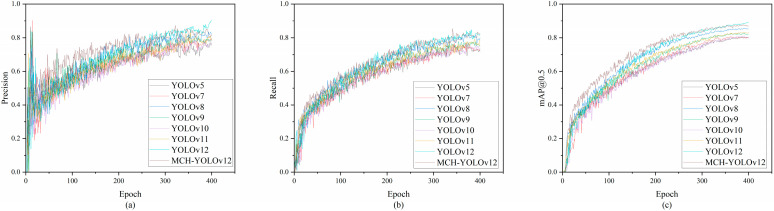
Visualization results of the comparative experiments. (**a**) The precision comparison chart of each model; (**b**) the recall comparison chart of each model; (**c**) the mAP@0.5 comparison chart of each model.

Based on this, we conducted comparative experiments on the NEU-DET dataset using several mainstream detectors, including YOLOv5 through YOLOv12, with the results shown in Table 10. Additionally, model performance was compared across mAP@0.5, precision, and recall metrics, as illustrated in Figure 20. MCH-YOLOv12 achieved superior performance in all metrics, attaining the highest mAP@0.5 while maintaining high precision and recall, demonstrating consistent detection advantages. These results indicated that the proposed model possessed strong robustness and cross-domain generalization capability, effectively adapting to defect types beyond those in the original training domain.

As can be seen in the comparative experimental results in the table, our proposed model exhibited significant advantages across multiple key performance metrics. Specifically, our method outperformed all comparative models across four precision metrics: precision, recall, mAP@0.5, and mAP@0.5~0.95. In particular, it achieved a 6.0% improvement in mAP@0.5~0.95 compared to the state-of-the-art YOLOv12, demonstrating stronger comprehensive detection capability and generalization performance.

Furthermore, it exhibited excellent performance in maintaining low model complexity. Our method contained only 6.8 M parameters and 16.9 G FLOPs. In contrast, although YOLOv12 achieved relatively high accuracy, its parameter count and computational load were 10.7 M and 19.3 G, respectively. While YOLOv8 exhibited relatively close accuracy, its FLOPs were as high as 25.7 G. These results indicated that our method, while maintaining high accuracy, significantly reduced the model’s computational overhead and deployment costs, demonstrating superior lightweight advantages and higher practical application value.

**Figure 21:**
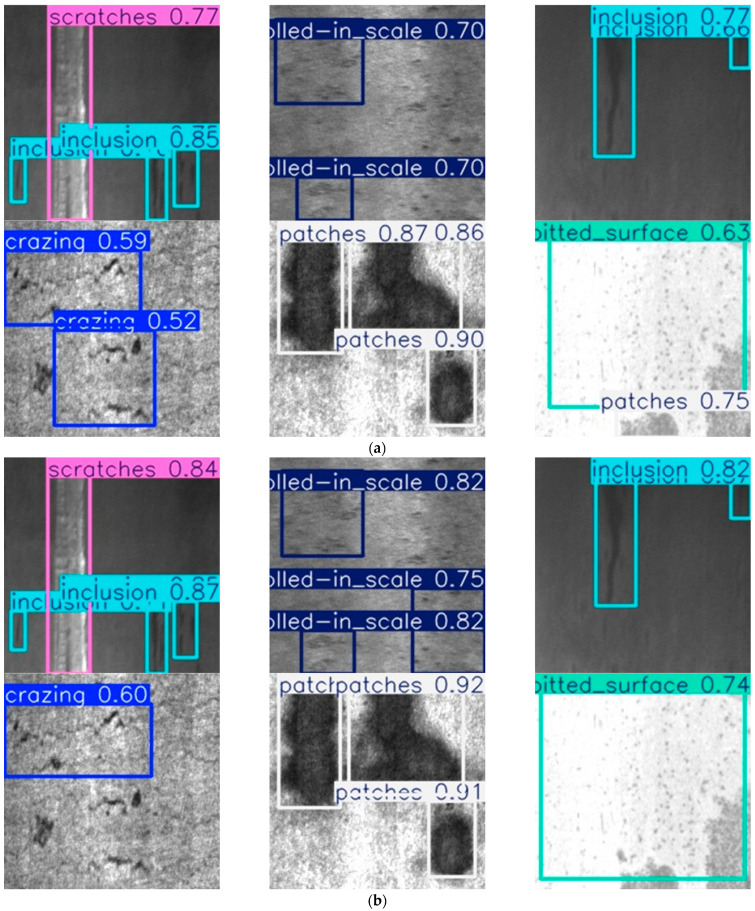
Performance of YOLOv12 and MCH-YOLOv12 on 6 types of surface defects in the NEU-DET dataset. (**a**) Detection results of the 6 defects in YOLOv12. (**b**) Detection results of the 6 defects in MCH-YOLOv12.

The detection results of six types of surface defects in the NEU-DET dataset, using YOLOv12 and MCH-YOLOv12, respectively, are shown in Figure 21.

The evaluation results on the NEU-DET dataset confirmed the strong generalization and cross-domain robustness of the proposed MCH-YOLOv12 model. It maintained superior detection accuracy across various defect types and imaging conditions, thereby validating its applicability to broader industrial surface inspection tasks.

### 4.8. Analysis of Failure Cases

**Figure 22:**
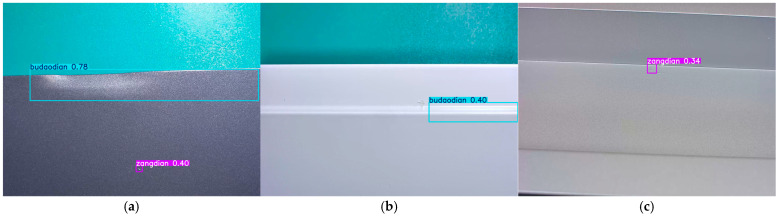
Failure cases. (**a**) Aoxian is misclassified as a budaodian. (**b**) Tufen is missed (no detection output). (**c**) Pengshang is misclassified as a zangdian.

To conduct a more comprehensive evaluation of the proposed model, we analyzed representative failure cases from the test set (as illustrated in Figure 22). These cases cover misdetections and missed detections of surface defects on aluminum profiles, enabling us to identify the model’s performance bottlenecks in complex scenarios.

The causes of detection failures can be summarized as follows: concave defects and non-conductive defects exhibit high similarity in grayscale distribution and edge blurriness, making it difficult for the model to accurately distinguish their morphological features and thus leading to misclassification; convex powder defects, due to their tiny size and low contrast against the background, have weak feature signals that fail to be effectively captured by the model, resulting in missed detections; scratches and dirty spots share certain commonalities in local texture performance, and the model’s insufficient extraction of fine-grained differential features between them leads to category confusion and misclassification. These failure cases reflect that the model still has room for improvement in terms of fine-grained modeling of morphological features and the ability to enhance differential features when dealing with low-contrast, small-sized, and visually similar defects.

To address the issues exposed by the aforementioned detection failures, future model optimization will proceed in three directions: first, designing a morphology-aware attention mechanism that incorporates geometric prior features such as curvature and circularity to strengthen the modeling of shape characteristics for defects like aoxian and pengshang, thereby reducing category confusion caused by visual similarity; second, constructing a multi-scale weak feature enhancement module to improve the capture capability of weak signals for small-sized, low-contrast defects (e.g., tufen) through cross-layer feature aggregation and adaptive threshold adjustment, aiming to lower the missed detection rate; third, expanding the dataset with edge cases under extreme lighting and complex backgrounds, combined with generative data augmentation techniques, to enhance the model’s generalization ability in complex industrial scenarios. Ultimately, this will enable more accurate and robust detection of various surface defects on aluminum profiles, providing more reliable technical support for industrial quality inspection.

## 5. Conclusions

We proposed the MCH-YOLOv12 algorithm to address the challenges of surface defect detection in aluminum profiles. By introducing multiple innovative technologies and optimization strategies, we significantly improved the model’s detection accuracy and robustness. Experimental results on the aluminum profile surface defect dataset showed that the MCH-YOLOv12 algorithm achieved significant performance metrics in precision, recall, mAP@0.5, and mAP@0.5~0.95. Compared to the original YOLOv12 algorithm, it achieved improvements of 7.9%, 3.2%, 3.5%, and 2.5% in these metrics, respectively, while parameters and FLOPs were reduced by 7.0 M and 2.5 G, respectively. The improved algorithm showed significant enhancements across all performance metrics, demonstrating the effectiveness of the algorithm’s refinements. Additionally, the algorithm demonstrated high practicality and scalability, making it widely applicable in various industrial sectors, such as metal processing, automotive manufacturing, and aerospace.

## Figures and Tables

**Table 1:** Symbol explanation.

Symbol	Definition
X	input feature map
X_p_	the base feature map output by the main convolutional module
X_gi_	the multi-scale feature map generated by the *i*-th branch
Y	the final fused feature map
C	the number of channels in the input feature map
C’	the target number of channels for the final feature map
m	the number of output channels of the main convolutional module
H,W	the height and width of the feature map
W_p_	parameters of the 1 × 1 convolutional kernel in the main convolutional module
W_gi_	the parameters of the depthwise separable convolution kernel for the *i*-th branch
k_i_	the kernel size of the *i*-th branch
s	the reduction ratio of the main convolution module
n	the number of branches in the multi-scale derivation module
BN	Batch Normalization
ReLU	Rectified Linear Unit
Concat	channel concatenation operation

**Table 2:** Comparison of SCCGLU with representative attention mechanisms.

Attention Module	Attention Type	Enhanced Dimensions	Key Mechanism	Complexity	Suitability for Defect Detection
SE-Net	Channel-wise	Channel	Squeeze-and-Excitation	Low	Good for global context; lacks spatial focus
CBAM	Channel and Spatial	Channel and Spatial	Channel and spatial attention in sequence	Medium	Better local focus; still limited on edges
SA	Self-Attention	Full Feature Map	Long-range dependencies with high computational cost	High	Powerful but expensive for real-time use
GAM	Global Attention	Global Channel + Spatial	Global context fusion with separate attention branches	High	Effective but overcomplex for light models
SCCGLU (Ours)	Direction-aware and Edge	Directional Channel + Edge Spatial	Combines directional channel modeling and edge-aware enhancement via Gated Linear Unit	Medium	Designed for fine-grained, edge-blurred, irregular defects

**Table 3:** Comparison of loss functions.

Loss Function	Applied Module	Purpose	Key Feature
CIoU Loss	Anchor-based regression	Optimizes bounding box localization by considering overlap, center distance, and aspect ratio	More accurate and stable than IoU and GIoU, especially in tight bounding box regression
BCE Classification Loss	Anchor-based classification	Binary classification to predict object presence per anchor	Simple cross-entropy loss; does not address class imbalance
GIoU Loss	Anchor-free regression	Optimizes bounding box regression by penalizing non-overlapping predictions	Improves over IoU when predicted boxes do not overlap with ground truth
Focal Loss	Anchor-free classification	Enhances classification by addressing foreground–background imbalance	Down-weights easy examples, focuses on hard examples, mitigates class imbalance
BCE Centerness Loss	Centerness prediction	Supervises the centerness score, indicating how close a point is to object center	Helps suppress low-quality predictions far from the center of objects

**Table 4:** A comparison of structural components between YOLOv12 and MCH-YOLOv12.

Module	YOLOv12	MCH-YOLOv12	Purpose of Modification	Benefit
Backbone	Standard convolution	MultiScaleGhost Conv replace Standard Conv	To overcome the limitation of single-scale feature extraction	Enhance feature representation and detection of irregular defects
Neck	C3k2	SCCGLU-C3k2: Spatial-Channel Collaborative Gated Linear Unit added after C3k2	To better capture direction-aware and edge-sensitive features	Improve perception of fine-grained or edge-localized defect features
Head	Anchor-based	Hybrid Head: combines anchor-based and anchor- free branches	To adapt to varied object shapes/sizes and handle class imbalance	Improve accuracy, robustness, and localization under diverse scenarios

**Table 5:** Experimental environment.

Name	Experimental Configuration
Programming language	Python 3.12
Deep learning framework	PyTorch 2.3.0 + CUDA 12.1
CPU	Intel (R) Xeon (R) Platinum 8352 V (Intel Corporation, Santa Clara, CA, USA.)
Memory	48 GB
GPU	RTX 3080x2 (20 GB) (NVIDIA Corporation, Santa Clara, CA, USA.)
Development environment	JupyterLab (version 3.6.3)

**Table 6:** Parameter settings.

Parameter	Parameter Value
Input image size	640 × 640
Number of CPU threads	8
Initial learning rate	0.01
Final learning rate	0.01
Batch size	32
Optimizer	SGD
Number of training rounds	400

**Table 7:** Comparison of detection results between MCH-YOLOv12 and YOLOv12.

Models	jupi	budaodian	tufen	cahua	aoxian	qikeng	zangdian	tucengkailie	loudi	pengshang	mAP@0.5/%
YOLOv12n	94.0	86.9	88.5	88.6	86.2	65.4	76.0	93.1	76.9	93.8	91.5
Ours	98.6	95.0	92.7	95.9	92.1	73.6	82.7	99.2	86.7	94.3	95.0

**Table 8:** Ablation experiment results.

Model Number	MultiScaleGhost	SCCGLU-C3K2	Hybrid Head	Precision/%	Recall/%	mAP@0.5/%	mAP@0.5~0.95/%	Parameter/10^6^	FLOPs/G
A0	✗	✗	✗	84.9	87.9	91.5	69.4	11.1	19.6
A1	√	✗	✗	87.2	88.7	92.9	71.6	8.3	18.3
A2	✗	√	✗	86.1	91.1	93.6	74.1	7.5	19.4
A3	✗	✗	√	90.3	84.9	92.3	71.6	7.1	18.2
A4	√	√	✗	87.0	89.7	93.3	71.7	9.3	18.8
A5	√	✗	√	88.6	89.5	94.1	74.3	9.4	18.6
A6	✗	√	√	89.5	89.8	94.3	75.6	8.0	19.0
A7	√	√	√	92.8	91.1	95.0	71.9	7.0	17.1

**Table 9:** Evaluation of Experimental Outcomes.

Model	Precision/%	Recall/%	mAP@0.5/%	mAP@0.5~0.95/%	Parameter/10^6^	FLOPs/G
YOLOv5	81.3	83.4	87.2	62.0	9.6	16.5
YOLOv7	83.9	86.0	90.1	66.7	36.9	104.7
YOLOv8	86.6	84.5	91.6	69.1	11.2	28.6
YOLOv9	72.2	67.3	72.7	46.0	9.1	26.7
YOLOv10	84.1	81.4	87.8	64.5	9.3	21.6
YOLOv11	83.9	82.2	88.0	65.1	9.4	21.5
YOLOv12	84.9	87.9	91.5	69.4	11.1	19.6
Faster-RCNN	88.6	89.5	94.1	74.3	41.3	251.4
SSD	84.6	77.4	85.5	60.2	24.7	98.3
Ours	92.8	91.1	95.0	71.9	7.0	17.1

**Table 10:** Comparison of experimental results.

Model	Precision/%	Recall/%	mAP@0.5/%	mAP@0.5~0.95/%	Parameter/10^6^	FLOPs/G
YOLOv5	76.7	72.8	80.3	54.1	8.7	15.3
YOLOv7	77.9	72.8	80.4	55.4	33.4	98.7
YOLOv8	81.3	80.4	85.2	61.4	10.8	25.7
YOLOv9	78.5	76.5	82.3	58.0	8.3	24.5
YOLOv10	78.0	71.6	81.1	54.0	8.9	20.1
YOLOv11	78.3	78.8	83.1	58.3	8.6	20.7
YOLOv12	85.0	80.3	87.9	61.4	10.7	19.3
Ours	89.6	82.0	89.2	67.4	6.8	16.9

## Data Availability

The datasets presented in this article are not readily available because the data are part of an ongoing study. Requests to access the datasets should be directed to 231502546@mails.ccu.edu.cn.
